# Mental Health of Homicidally Bereaved Individuals: A Systematic Review of Post-Homicide Factors

**DOI:** 10.1177/00302228241245751

**Published:** 2024-04-07

**Authors:** Sarah Lebel, Olivier Lépine, Pascale Brillon

**Affiliations:** 1 Psychology Department, 14845 Université du Québec à Montréal, Montreal, QC, Canada

**Keywords:** homicide, bereavement, adults and death, grief, loss

## Abstract

Experiencing the homicide of a loved one has a substantial impact on the mental health of family members and friends who must survive their loved one’s tragic death. This systematic review aims to synthesize the current findings on post-homicide factors and identify the factors most frequently related to the mental health of homicidally bereaved individuals (HBI). Four databases were searched (PsycINFO, SCOPUS, Sociological Abstract, PubMed). The selection of studies was based on a peer review process conducted by two independent researchers to ensure interrater reliability. The articles were screened to ensure the presence of homicidally bereaved adults, resulting in a total of 35 eligible papers to be considered in the current review. Factors were organized into categories, with the criminal justice system-related factors (*n* = 18), social factors (*n* = 17), and coping factors (*n* = 13) being the most prevalent. This review identifies clinical avenues for preventing distress and fostering the well-being of HBI.

Losing a loved one to homicide is a tremendously disturbing experience (Kristensen et al., 2012). It has been estimated that for each homicide, seven to ten individuals close to the victim are negatively affected in several areas of their life (Redmond, 1989). They may experience difficulties returning to work, adjusting to new roles, such as becoming a widowed parent, face financial burdens, or be required to attend criminal justice proceedings (Connolly & Gordon, 2015). In addition to these consequences, a systematic review has shown that homicidally bereaved individuals (HBI) report high levels of posttraumatic stress (PTS) and prolonged grief (PG) symptoms (van Denderen et al., 2015). In fact, lifetime prevalence of PTS disorder among the HBI significantly surpasses that of the general population, with occurrences ranging from 19.1% to 71%, compared to just 2.4%–3.5% in the general population (Van Ameringen et al., 2008). The DSM-5-TR criteria for PG disorder emphasizes the presence of intense yearning or longing for the deceased, as well as preoccupation with thoughts or memories of the deceased. A recent study demonstrated that 34.3% of their sample of bereaved individuals met PG disorder criteria (Thieleman et al., 2023). Moreover, mental health problems are markedly more severe among HBI than other bereaved populations, such as those bereaved by suicide, accident, or nonviolent death (Boelen et al., 2015; Murphy et al., 1999, 2003). In addition, findings by Simmons et al. (2014) indicate that the well-being of HBI may also be significantly impaired. In fact, results from this study highlighted that HBI reported relatively low levels of satisfaction with their lives. The posttraumatic growth (i.e., significant positive change following challenging life circumstances; Tedeschi & Calhoun, 1995) of young adults who have lost a loved one to homicide, accident, or suicide (*M* = 57.5; Currier et al., 2013) has also shown to be quite low in comparison to other traumatized individuals, such as female victims of intimate partner violence (*M* = 68.1; Cobb et al., 2006). In order to develop a more complete understanding of mental health, the dual-continua or dual-factor models of mental health (Keyes, 2005; Massé et al., 1998) emphasize the importance of considering positive (e.g., well-being) and negative (e.g., distress) dimensions of mental health as distinct constructs, rather than two ends of a single spectrum. That is, mental health is not uniquely the absence of distress but includes the presence of positive psychological states, such as well-being.

Previous studies have revealed significant variations among HBI in the intensity of their psychological distress. Therefore, it appears imperative for researchers to identify the factors responsible for these variations. To this end, three types of factors clarify the underlying explanatory processes of mental health in trauma victims (Sayed et al., 2015). First, *peritraumatic factors* refer to factors related to the traumatic event itself, such as its characteristics, the circumstances (e.g., witnessing the homicide), and the emotions experienced during the event, or in the days surrounding it (e.g., experience of helplessness). Second, *pretraumatic factors* are those present prior to the traumatic event. These refer to factors such as the quality of the relationship with the deceased. Third, *posttraumatic factors* occur after the trauma and can affect cognition, such as the beliefs an individual holds about the world. Posttraumatic factors are of particular interest as they are often malleable dimensions that can be targeted in therapy to promote resilience and reduce PTS symptoms (Bourdon, et al., 2019).

Joseph et al. (1995)’s Integrative Model of Psychosocial Factors Relating to Adaptation to Traumatic Stress presents key psychosocial factors that may influence posttraumatic distress. This model categorizes factors into three levels: *cognitive* (e.g., appraisals of the traumatic event), *coping* (e.g., religious coping strategies), and *social* (e.g., social support from a loved one). Moreover, *emotional factors* following the traumatic event can influence the appearance of later psychological states. For example, anger and rage are emotional states that could aggravate PTS symptoms (Riggs et al., 1992). Furthermore, unique experiences, such as interacting with the criminal justice system (CJS) following the loss of a loved one by homicide, could represent an additional stressful event (Gekoski et al., 2013). In recent years, researchers have been particularly interested in the potential deleterious effect of experiences with the CJS on the victim’s mental health. The literature has addressed this effect in various ways. Of particular interest is the *perception of justice* as it has been argued that perceptions of fairness can reduce the stress associated with uncertainty and help with the coping process (Van den Bos & Lind, 2002). To date, most frameworks in the scientific literature have focused on one specific dimension of the perception of justice, with the exception of Colquitt (2001). Colquitt identified four types of perceptions of justice that can impact effective organizational functioning: 1) distributive (i.e., quality of the decision outcomes); 2) procedural (i.e., quality and fairness of the procedures or formal rules); 3) interpersonal (i.e., quality of treatment by authorities); and 4) informational (i.e., quality of information provided by authorities). Along with the perception of justice, *satisfaction with the CJS* can uniquely contribute to the experience of victims in the CJS, and in turn, impact their mental health. For example, offering interventions that are voluntary and flexible, both in terms of objective and scheduling, has been shown to maintain victim satisfaction with the CJS (Van Camp & Wemmers, 2013), which to our knowledge, cannot be explained by current conceptualizations of perceptions of justice. This suggests that the level of satisfaction with the CJS involves more than just the perception of justice. Both Colquitt’s model and the level of satisfaction with the CJS help to highlight the contributions of unique factors, such as CJS-related factors, in the bereavement of homicide victims. The existing research indicates that these factors have a substantial impact on the mental health of adults exposed to traumatic events (e.g., military, natural disaster, criminal acts, accidents, and others; Boulware & Bui, 2016; Lens et al., 2015; Matthews & Marwit, 2004; Levi-Belz and Lilac Lev-Ari, 2019).

Previous systematic reviews dealt with various aspects of HBI, such as prevalence rates of distress symptoms in HBI (van Denderen et al., 2015), the impact of the homicide on their life (Connolly & Gordon, 2015), and their perception of the effectiveness of interventions (Alves-Costa, et al., 2021). However, they did not provide an overview of the current evidence concerning the role of post-homicide factors on the mental health of the bereaved. A systematic review of this particular topic could help to enlighten mental health professionals on critical post-homicide factors identified in the scientific literature, guiding them to develop more effective treatment strategies to mitigate distress and enhance the well-being of HBI. Therefore, the objective of this review is to summarize the current scientific literature on *post-homicide factors* (i.e., factors occurring after the homicide) that contribute to the mental health, namely distress and well-being, of HBI using a narrative synthesis based on the production standards of Dagenais et al. (2013).

## Method

### Search Strategy and Study Selection

Specific keywords were systematically searched in the following four databases regardless of date or language: PsycINFO, SCOPUS, Sociological Abstract, and PubMed. The following keywords were entered into each database: (homicid* OR murder OR manslaughter OR assassination) AND (surviv* OR “co-victim*” OR “covictim*” OR “secondary victim*” OR bereave*). To ensure a large enough scope in which all mental health indicators could be included, no keywords were specified in the search string concerning mental health. Three additional searches were conducted: 1) searches for the keywords using Google Scholar; 2) manual searches in the reference lists of three systematic reviews about HBI; and 3) establishing contact with the first author of the included articles to inquire about unpublished research or known eligible articles. Four additional articles were identified using this strategy. This systematic search was completed in December 2021. Following the Preferred Reporting Items for Systematic Reviews and Meta-Analyses (PRISMA) guidelines, the protocol for this review was registered in Prospero [registration number; CRD42022234219].

Five inclusion criteria were established to determine the eligibility of the articles: 1) the inclusion of only homicidally bereaved individuals; 2) being 18 years of age or older; 3) conducting one statistical or qualitative analysis, between at least one post-homicide factor and one mental health indicator; 4) the availability of the full-text article, publication in a peer-reviewed journal and an empirical research design (i.e., quantitative, qualitative or mixed methods); and 5) the article not solely measuring intervention effectiveness.

### Data Collection and Extraction

*EndNote 20.2* software was used throughout the screening process. All duplicate articles were removed. All keywords, titles, and abstracts obtained were independently screened by two evaluators and articles that did not match the inclusion criteria were excluded. The full text of the remaining articles were reviewed and both evaluators’ ratings of the five criteria were compared. In the event of discrepancies between evaluators, a discussion ensued until a consensus was reached. For each study, the following data was extracted by both evaluators: study design and characteristics (e.g., time since loss), post-homicide factors, and mental health indicators. Results related to the association between post-homicide factors and mental health indicators were also extracted. Once again, any discrepancies in this process were discussed until a consensus was reached.

### Risk of Bias Assessment

The Appraisal tool for Cross-Sectional Studies (AXIS; Downes et al., 2016) was used to assess the risk of bias of articles. This AXIS is a validated 20-item scale which provides a total score by assessing the validity and reliability of each step in a scientific study (i.e., introduction, method, results, discussion, other) with a three-point scale (0 = *No*; 1 = *Yes*; 2 = *I don’t know*). A score of 1 is given for all items where “yes” is endorsed and a score of zero for items where “no” or “I don’t know” are endorsed. The total score ranges from zero to 20, with higher scores indicating a lower risk of bias. Articles were reviewed using the AXIS tool and the evaluators’ ratings were compared. When discrepancies were identified, a discussion among the evaluators took place until a consensus was reached. Inter-rater agreement between both evaluators was assessed to evaluate reliability.

The risk of bias assessment of our selected articles revealed a minimum score of 7/20 and a maximum score of 19/20 with a mean score of 14.9/20 (74.6%). Inter-rater reliability for the risk of bias analysis was 87.1% (agreement on 610 items out of 700) with a kappa of 0.70, which is consistent previous studies (88.9%; Downes et al., 2016). Most studies included in the current review recruited participants from community-based organizations in the United States and included measures of self-report and instruments developed specifically for their study.

## Results

**Figure 1.**
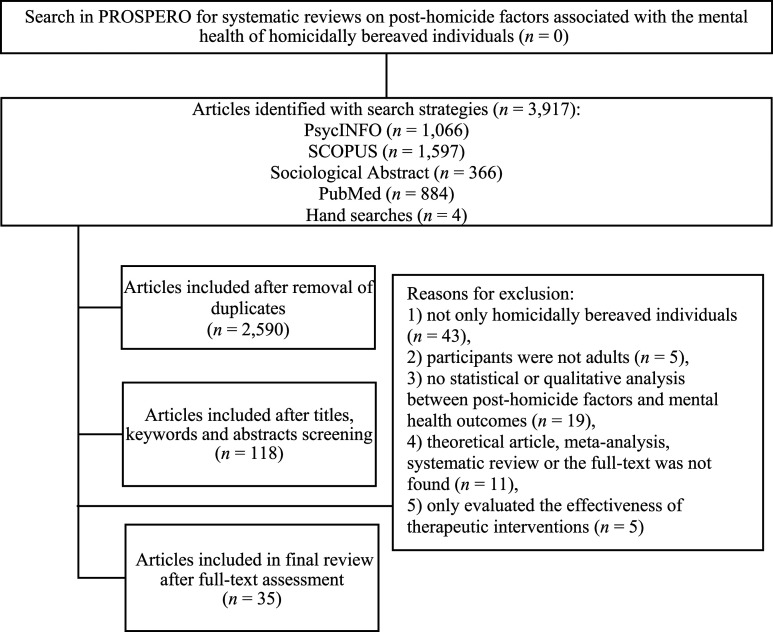
Flowchart of the search process, adapted from the recommendations of the PRISMA guidelines.

The database search identified 3913 articles, with an additional 4 articles identified in the reference lists and Google Scholar (total of 3917 articles). As shown by Figure 1, 2590 articles were included after the removal of duplicates; 118 were included after the titles, keywords, and abstracts screening; and 35 met full inclusion criteria after a full-text analysis. All 35 articles were written in English. Of the 35 articles, 26 were cross-sectional in design (18 quantitative studies, 8 qualitative studies), 7 were longitudinal (1 qualitative study, 6 quantitative studies, with the time point ranging between 2 and 5), and 2 were mixed. Moreover, 22 articles included participants from the United States, four from the United Kingdom, four from the Netherlands, two from Canada, and three articles did not mention such information. A total number of 4688 HBI were studied (*M* = 130). Participants were 49.1 years old on average and the overall age range was from 18 to 86 years old. The average time elapsed since the loss was 4.4 years.

Measures used to assess post-homicide factors and mental health varied widely (e.g., self-report instruments, clinical interviews, questions developed by the authors). As illustrated in Table 1, post-homicide factors (regardless of significance) associated with mental health indicators were organized into six categories based on the works of Joseph et al. (1995) and Colquitt (2001): cognitive factors, coping factors, social factors, emotional factors, CJS-related factors, and other factors. The categories of factors are presented from the most prevalent to the least prevalent. In accordance with our objectives, negative and positive dimensions of mental health were organized into distress and well-being.

**Table 1.** Characteristics, Results, and Quality Assessments of Included Studies (*n* = 35).

First author (year)	*N*	Post-homicide factor	Direction of association	Mental health outcome		Risk of bias
CJS-related factors (*n* = 18)						
Quantitative studies						
Amick-McMullan (1989)	19	Satisfaction with the CJS	Negative	PTS and anxiety symptoms, psychological maladjustment	D	12
Boelen (2016)	331	Having a suspect being identified, arrested, or convicted	Negative	PG symptoms	D	18
			*ns*	Anger, revenge, PTS symptoms		
Johnson (2021)	112	Perceived justice	*ns*	Posttraumatic growth	W	18
Milman (2018)	47	Perpetrator being arrested	Positive	Major depression	D	16
			*ns*	PTS and PG disorder		
		Satisfaction with the police	Positive	PG disorder		
		Satisfaction with the CJS	Positive	Major depression		
Simmons (2014)	137	Ongoing prosecution or investigation, the perpetrator not being caught	Positive	PTS symptoms	D/W	11
		Case status or case closure	*ns*	Life satisfaction		
Soydas (2020)	929	Having the verdict held during therapy	Negative	PTS and PG symptoms	D/W	18
			Positive	General adjustment		
			*ns*	Anxiety and depressive symptoms		
		Having no verdict held during therapy	Negative	PTS symptoms		
			*ns*	PG, anxiety, and depressive symptoms, general adjustment		
Soydas (2021)	923	Having the verdict held before therapy	*ns*	Distress and depressive symptoms	D	16
Thompson (1996)	150	Police satisfaction, being informed about the case, arrest status	*ns*	Distress symptoms (i.e., depression, anxiety, somatization, hostility)	D	14
van Denderen (2014)	331	Juridical status of the perpetrator	*ns*	Dispositional revenge, situational revenge	D	16
van Denderen (2016)	312	Ongoing legal process	Positive	CG and PTS symptoms	D	16
Williams (2015)	47	Satisfaction with the police	Negative	Depressive symptoms	D	17
		Satisfaction with other CJS departments	*ns*	Depressive, PTS, and CG symptoms		
**Mixed studies**						
Van Wijk (2017)	62	Ongoing criminal proceedings	Positive	Anger, grieving symptoms, emotional problems	D	14
**Qualitative studies**						
Alves-Costa (2018)	29	Ongoing criminal proceedings	Positive	Inability to fully grieve	D	16
Alves-Costa (2021)	21	Lack of information from the CJS	Positive	Stress, maladjustment	D	16
Baliko (2008)	10	Dissatisfaction with the CJS	Positive	Anger	D	17
		Perception of justice	Negative	Anger		
Stretesky (2010)	37	Non-prosecution of the case	Positive	Anger, despair, grieving symptoms	D/W	17
		Stopped communication with CJS	Negative	Sense-making		
		Lack of knowledge of detectives	Positive	Stress related to the loss		
Thiel (2016)	15	Experience with the CJS	Positive	Grieving symptoms	D	12
		Lack of information about the case	Positive	Grieving symptoms		
		Acquittal	Positive	Anger, grieving symptoms		
Wellman (2014)	24	Failure to apprehend a suspect	Positive	Anger	D	17
**Social factors (*n* = 15)**						
**Quantitative studies**						
Rheingold and Williams (2015)	47	Perceived social support following the death of a loved one	Negative	Major depression, PTS disorder	D	17
			*ns*	CG		
Rynearson (1995)	50	Seeking treatment	Positive	Bereavement and PTS symptoms, dissociation, reenactment	D	10
Soydas (2020)	929	Having a funeral held during therapy	Positive	PTS symptoms	D/W	18
			*ns*	PG, depressive, and anxiety symptoms, general adjustment		
		Having no funeral held during therapy	Positive	PG symptoms		
			*ns*	PTS, depressive, and anxiety symptoms, general adjustment		
Soydas (2021)	923	Having a funeral held before therapy	Positive	Distress and depressive symptoms	D	16
Sprang (1998)	171	Social support	Negative	PTS and grieving symptoms	D	12
Thompson (1998)	150	Parental and economic role changes	Positive	PTS and distress symptoms	D	14
		Marital role changes	*ns*	PTS and distress symptoms		
Williams (2015)	47	Barriers to accessing services	Positive	Depressive symptoms	D	17
			*ns*	PTS and CG symptoms		
**Mixed studies**						
Bottomley (2017)	47	Need for physical assistance	Positive	Grief symptoms	D	14
			*ns*	Depressive, PTS, and anxiety symptoms		
		Satisfaction with physical assistance	Negative	Anxiety and PTS symptoms		
			*ns*	Depressive and grief symptoms		
Burke (2010)	54	% Negative relationships	Positive	Dysfunction in bereavement, PTS symptoms	D	14
			*ns*	Depressive symptoms		
		Having an available support network	Negative	Dysfunction in bereavement		
			*ns*	PTS and depressive symptoms		
		Grief support	Negative	Depressive symptoms		
			*ns*	Dysfunction in bereavement, PTS symptoms		
		Perceived need for and satisfaction with social support	*ns*	Dysfunction in bereavement, PTS and depressive symptoms		
		Advice needs, % family/non-family support, % anticipated negative relationships	*ns*	Dysfunction in bereavement, depressive, and PTS symptoms		
Thompson (1996)	150	Perceived social support	Negative	Depressive symptoms, hostility	D	14
			*ns*	Anxiety symptoms, somatization		
Van Wijk (2017)	62	Reminders of the loss	Positive	Grieving symptoms	D	14
**Qualitative studies**						
Alves-Costa (2018)	29	Reminders of the loss, being active on social media platforms	Positive	Maladjustment, anxiety symptoms, emotional pain	D	16
Alves-Costa (2021)	21	Financial changes	Positive	Distress symptoms	D	16
Hannays-King (2015)	10	Difficulties with intimate relationships, lack of social support	Positive	Stress symptoms	D	11
		Stigma of gun violence	Positive	Shame		
Wellman (2014)	24	Family support	Negative	Grieving symptoms	D	17
**Coping factors (*n* = 14)**						
**Quantitative studies**						
Boelen (2016)	331	Depressive avoidance	Positive	PTS and PG symptoms	D	18
			*ns*	Anger, revenge		
		Anxious avoidance	Positive	Revenge		
			*ns*	Anger, PTS, and PG symptoms		
Burke (2011)	46	Negative religious coping	Positive	Dysfunction in bereavement	D	17
		Positive religious coping	*ns*	Dysfunction in bereavement		
Johnson (2021)	112	Being christian	Positive	Posttraumatic growth	W	18
McDevitt-Murphy (2021)	54	Hazardous drinking	Positive	PTS, depressive, and CG symptoms	D	19
Neimeyer (2011)	46	Negative religious coping	Positive	Grief, PTS, and depressive symptoms	D	17
		Positive religious coping	*ns*			
Simmons (2014)	137	Visiting the grave	Positive	PTS symptoms	D/W	11
		Being active in church, spending time with loved ones, not drinking alcohol	Positive	Life satisfaction		
Sprang (1998)	171	Having religious beliefs	Negative	PTS and grieving symptoms	D	12
Thompson (1997)	150	Pleading, discontent with god	Positive	PTS and distress symptoms	D	13
		Deeds	Positive	Distress symptoms		
		Religious support	Negative	Distress symptoms		
		Religious support, deeds, spiritually based coping, avoidance	*ns*	PTS symptoms		
		Spiritually based coping, avoidance	*ns*	Distress symptoms		
Tuck (2012)	8	Behavioral avoidance, positive coping, negative coping	ns	Distress (i.e., PTS and depressive symptoms, responses to traumatic events, grief reactions), well-being (i.e., general/spiritual well-being)	D/W	17
**Mixed studies**						
-	-	-	-	-	-	-
**Qualitative studies**						
Baliko (2008)	10	Resilience	Positive	Personal growth	W	17
Hannays-King (2015)	10	Avoidance	Positive	Grieving symptoms	D	11
			Negative	Grieving symptoms		
Kenney (2003)	145	Balance in coping strategies	Negative	Alcohol and drug use, anger, suicidal ideation	D	7
Parappully (2002)	16	Helping others, spirituality, continuing bond with the victim, finding time to be alone	Positive	Growth, healing, joy, life satisfaction	W	12
Wellman (2014)	24	Distraction through work, having faith in religion	Negative	Grieving symptoms, emotional pain, anger, fear	D	17
**Cognitive factors (*n* = 7)**						
**Quantitative studies**						
Boelen (2016)	331	Catastrophic misinterpretations of grief reactions	Positive	PG symptoms	D	18
			*ns*	PTS symptoms, anger, revenge		
		Negative cognitions about the self	Positive	Anger		
			*ns*	PTS and PG symptoms, revenge		
		Negative cognitions about the future	Positive	Revenge		
			*ns*	PTS and PG symptoms, anger		
		Negative cognitions about life	*ns*	PTS and PG symptoms, anger, revenge		
Johnson (2021)	112	Meaning-making	Positive	Posttraumatic growth	W	18
Thompson (1996)	150	Self-esteem	Negative	Distress symptoms	D	14
		Perception of control	Negative	Depressive and anxiety symptoms		
			*ns*	Somatization, hostility		
Tuck (2012)	8	Forgiveness, hope	*ns*	Distress symptoms, well-being	D/W	17
Zakarian (2019)	57	Meaning making	Negative	CG and PTS symptoms	D	14
**Mixed studies**						
-	-	-	-	-	-	-
**Qualitative studies**						
Kenney (2003)	145	Rigid gender roles	Positive	Depressive symptoms	D	7
Parappully (2002)	16	Acceptance of the loss, finding meaning	Negative	Emotional pain, feelings of rage	D/W	12
		Thankfulness	Positive	Personal growth		
**Emotion factors (*n* = 2)**						
**Quantitative studies**						
Tuck (2012)	8	Revenge	*ns*	Distress symptoms, well-being	D/W	17
van Denderen (2014)	331	Situational revenge	Positive	PTS symptoms	D/W	16
			Negative	Positive functioning		
		Dispositional and situational revenge	Positive	CG symptoms		
		Dispositional revenge	*ns*	PTS symptoms, positive functioning		
**Mixed studies**						
-	-	-	-	-	-	-
**Qualitative studies**						
-	-	-	-	-	-	-
**Other factors (*n* = 20)**						
**Quantitative studies**						
Amick-McMullan (1989)	19	Time since loss	*ns*	PTS symptoms, psychological maladjustment	D	12
Boelen (2016)	331	Time since loss	*ns*	PTS and PG symptoms, revenge	D	18
Burke (2011)	46	Time since loss	*ns*	Dysfunction in bereavement	D	17
Johnson (2021)	112	Time since loss	Positive	Posttraumatic growth	W	18
McDevitt-Murphy (2012)	54	Time since loss	Negative	Anxiety and PTS symptoms, dysfunction in bereavement	D	16
			*ns*	Depressive symptoms		
Milman (2018)	47	Employment status	Positive	Major depression	D	16
Neimeyer (2011)	46	Time since loss	Negative	Grief symptoms	D	17
			*ns*	Depressive and PTS symptoms		
Rheingold and Williams (2015)	47	Being employed full-time	Positive	Major depression, PTS disorder	D	17
			*ns*	CG		
Simmons (2014)	137	Working more hours	Positive	Life satisfaction	W	11
Sprang (1998)	171	Perceived health	Negative	PTS and grieving symptoms	D	12
		Time since loss	*ns*	PTS and grieving symptoms		
Soydas (2020)	929	Time since loss	Negative	PTS and PG symptoms	D/W	18
			*ns*	Depressive and anxiety symptoms, general adjustment		
Soydas (2021)	923	Time since loss	Negative	Distress and depressive symptoms	D	16
Thompson (1996)	150	Time since loss	*ns*	Distress symptoms	D	14
Thompson (1998)	150	Experiencing trauma(s) since the murder	Positive	Distress symptoms	D	14
		Time since loss	*ns*	PTS and distress symptoms		
Tuck (2012)	8	Time since loss	*ns*	Distress symptoms, well-being	D/W	17
Van Denderen (2014)	331	Time since loss	*ns*	Dispositional revenge	D	16
Van Denderen (2016)	312	Time since loss	Negative	CG and PTS symptoms	D	16
Williams (2012)	47	Physical health	*ns*	Grief, depressive, and PTS symptoms	D	17
		Time since loss	Negative	Grief and depressive symptoms		
			*ns*	PTS symptoms		
Williams (2015)	47	Satisfaction with services for homicide victims	Negative	Depressive symptoms	D	17
			*ns*	PTS and CG symptoms		
**Mixed studies**						
Van Wijk (2017)	62	Time since loss	Negative	Distress symptoms	D	14
**Qualitative studies**						
*-*	*-*	*-*	*-*	*-*	*-*	*-*

Note. D = distress; W = well-being; D/W = distress and well-being; CJS = criminal justice system; PTS = posttraumatic stress; PG = prolonged grief; CG = complicated grief; - = information not mentioned or absence of information.

### Criminal Justice System (CJS) Factors and the Mental Health of HBI

CJS-related factors represent the most documented category in the existing research (*n* = 18/35). This category is further divided into three subcategories: 1) events associated with the CJS; 2) perceptions of justice; and 3) satisfaction with the CJS.

#### CJS-Factors and Distress

For the subcategory Events Associated with the CJS, *ongoing criminal and legal proceedings* was linked to greater PTS and grieving symptoms as well as anger (Alves-Costa et al., 2018; Simmons et al., 2014; Thiel, 2016; van Denderen et al., 2016; van Wijk et al., 2017). *Acquittal* was also associated with greater anger and grieving symptoms (Thiel, 2016). Mixed results were observed for the *arrest status of the perpetrator*. In three studies, the non-prosecution of the case (Stretesky et al., 2010) and the perpetrator not being caught (Simmons et al., 2014; Wellman, 2014) were associated with more anger, despair, grieving and PTS symptoms. Similarly, Boelen et al. (2016) found that the identification, arrest, or conviction of a suspect was significantly linked to fewer PG symptoms, though it showed no associations with anger, revenge, or PTS symptoms. Furthermore, Milman et al. (2018) found that while the perpetrators’ arrest was positively linked to major depression, it was not associated with PTS nor PG disorder. Two other studies indicated that desire for revenge (van Denderen et al., 2014) and symptoms of distress, namely depression, anxiety, somatization and hostility (Thompson et al., 1996), did not differ significantly as a function of the arrest status of the perpetrator.

For the subcategory Perception of Justice, *lack of information regarding the CJS* was found to be associated with worse psychological adjustment, stress and grieving symptoms (Alves-Costa, Hamilton-Giachritsis, & Halligan, 2021; Thiel, 2016). *Lack of knowledge of detectives* further served to exacerbate feeling of stress related to the loss (Stretesky et al., 2010). However, one study revealed that being informed about the case was not significantly related to distress symptoms (Thompson et al., 1996). Overall *perception of justice* was linked to less anger (Baliko & Tuck, 2008).

For the subcategory Satisfaction with the CJS, mixed results were observed. One study reported positive and significant associations of *satisfaction with the CJS* with PG disorder and major depression (Milman et al., 2018). In contrast, in three other studies, *satisfaction with the CJS* (Amick-McMullan et al., 1989) and the police (Williams & Rheingold, 2015) were significantly linked to less depressive, PTS, and anxiety symptoms as well as better psychological adjustment. Further, *dissatisfaction with the CJS* was linked to more anger (Baliko & Tuck, 2008). Satisfaction with the police was not significantly associated with distress symptoms (Thompson et al., 1996).

#### CJS-Factors and Well-Being

For the subcategory Events Associated with the CJS, *having the verdict held during therapy* was significantly linked to better overall adjustment (Soydas et al., 2020). The *case being closed* did not show significant associations with life satisfaction (Simmons et al., 2014). For the subcategory Perception of Justice, *overall perceived justice* did not show significant associations with posttraumatic growth (Johnson, 2021). *Stopped communication with law enforcement* could negatively impact HBIs’ sense-making abilities (Stretesky et al., 2010).

### Social Factors and the Mental Health of HBI

Social factors were examined in 15 of the 35 included articles. This category is divided into two subcategories: 1) social context and 2) social support.

#### Social Factors and Distress

For the subcategory Social Context, *reminders of the loss* (Alves-Costa et al., 2018; van Wijk et al., 2017), *financial role changes* (Alves-Costa, Hamilton-Giachritsis, & Halligan, 2021; Thompson et al., 1998), *parental role changes* (Thompson et al., 1998), *seeking psychological treatment* (Rynearson, 1995), and *having a stigmatized status* (e.g., stigma of gun violence; Hannays-King et al., 2015) were linked to greater psychological maladjustment, dissociation, reenactment and shame, as well as increased PTS, anxiety, distress, and grieving symptoms. *Having a funeral* was also significantly linked to more PTS (Soydas et al., 2020), depressive and distress symptoms (Soydas et al., 2021). *Barriers to accessing services* was significantly linked to more depressive symptoms, though it was not associated with PTS or grieving symptoms (Williams & Rheingold, 2015). *Marital role change* was not significantly related to PTS or distress symptoms (i.e., depression, anxiety, somatization, hostility; Thompson et al., 1998).

For the subcategory Social Support, *being active on social media platforms* (Alves-Costa et al., 2018), *having negative relationships* (Burke et al., 2010), *having less social support* (Hannays-King et al., 2015), and *experiencing difficulties in relationships* (Hannays-King et al., 2015) were linked to worse maladjustment, as well as increased grieving, stress, and anxiety symptoms. Having an *available support network* (Burke et al., 2010) and *family social support* (Wellman, 2014) were linked to less depressive and grieving symptoms. Having more *grief support* (i.e., social support in bereavement) was also significantly linked to less depressive symptoms; however, it was not associated with dysfunction in bereavement nor PTS symptoms (Burke et al., 2010). Mixed results were observed for *perceived, need for, and satisfaction with social support*. Perceived social support was significantly associated with fewer grieving (Sprang & McNeil, 1998), and depressive symptoms and less hostility (Thompson et al., 1996). Moreover, the need for physical assistance was significantly linked to greater grieving symptoms (Bottomley et al., 2017). Perceived social support was not significantly associated with CG (Burke et al., 2010; Rheingold & Williams, 2015).

#### Social Factors and Well-Being

No study examined the role of social support in the well-being of HBI.

### Coping Factors and the Mental Health of HBI

Out of 35 studies, 14 addressed the coping category. This category is divided into two subcategories: 1) general coping strategies and 2) religious or spiritual coping strategies.

#### Coping Factors and Distress

For the subcategory General Coping Strategies, *visiting the grave* was significantly linked to more PTS symptoms (Simmons et al., 2014). Performance of *deeds* (i.e., attempts to live a better life following the event) was significantly associated with greater distress symptoms (i.e., depression, anxiety, somatization, hostility), though it was not associated with PTS symptoms (Thompson & Vardaman, 1997). *Hazardous drinking* was also significantly associated with more depressive, PTS, and CG symptoms (McDevitt-Murphy et al., 2021). *Finding a balance in coping strategies* was linked to lighter alcohol and drug use, less anger, and less suicidal ideation (Kenney, 2003). *Distraction through work* was also linked to fewer grieving symptoms, as well as less anger and fear (Wellman, 2014). Mixed results were observed for *avoidance strategies*. Boelen et al. (2016) showed that depressive avoidance (i.e., withdrawal from social, occupational, and recreational activities that could facilitate adjustment), but not anxious avoidance (i.e., avoidance of stimuli reminding the loss), was significantly linked to more PTS and PG symptoms. However, in two other studies, avoidance did not show significant associations with distress (i.e., PTS, grief, and depressive symptoms; Thompson & Vardaman, 1997; Tuck et al., 2012).

For the subcategory Religious or Spiritual Coping Strategies, *discontent with God* and *pleading*, which refers to passive dependence on God, were both significantly linked to greater distress and PTS symptoms (Thompson & Vardaman, 1997). *Negative religious coping* was also linked to more dysfunction in bereavement as well as greater PTS and depressive symptoms (Burke et al., 2011; Neimeyer & Burke, 2011). *Having religious beliefs* (Sprang & McNeil, 1998) and *faith in religion* (Wellman, 2014) were linked to less anger and fear, as well as fewer grieving and PTS symptoms. *Religious support* was significantly linked to less distress, though it was not associated with PTS symptoms (Thompson & Vardaman, 1997). Non-significant results were observed for *positive religious coping* and *spiritually-based coping and avoidance*. Positive religious coping was not significantly associated with PTS, depressive, nor grieving symptoms (Burke et al., 2011; Neimeyer & Burke, 2011; Tuck et al., 2012). Spiritually-based coping and avoidance were not significantly related to distress nor PTS symptoms (Thompson & Vardaman, 1997).

#### Coping Factors and Well-Being

For the subcategory General Coping Strategies, *continuing bond with the victim*, *finding time to be alone*, and *helping others* (Parappully et al., 2002) as well as *spending time with family and friends* and *not drinking alcohol* (Simmons et al., 2014) were linked to more personal growth, life satisfaction, and joy. *Resilience* (Baliko & Tuck, 2008) was also linked to more personal growth. Tuck et al. (2012) reported that *behavioral avoidance* was not significantly linked to well-being. For the subcategory Religious or Spiritual Coping Strategies, *spirituality* (Parappully et al., 2002), *being active in church* (Simmons et al., 2014), and *being Christian* (Johnson, 2021) were linked to more posttraumatic growth, life satisfaction, and joy.

### Cognitive Factors and the Mental Health of HBI

Out of 35 studies, seven covered cognitive factors. This category is divided into two subcategories: 1) appraisals of the traumatic event, and 2) cognitive style.

#### Cognitive Factors and Distress

For the subcategory Appraisals of the Traumatic Event, *negative grief* cognitions about the self and the future, as well as catastrophic misinterpretations, were significantly linked to greater PG symptoms, more anger, and revenge (Boelen et al., 2016). *Acceptance of the loss* (Parappully et al., 2002) and *meaning-making* (Parappully et al., 2002; Zakarian et al., 2019) were both associated with less feelings of rage, and fewer grieving and PTS symptoms. Negative cognitions about life were not significantly associated with PTS nor PG symptoms nor anger nor revenge (Boelen et al., 2016). Regarding *forgiveness specific to the loss*, Tuck et al. (2012) found no significant association with distress symptoms.

For the subcategory Cognitive Styles, *endorsement of rigid gender roles* was linked to more depressive symptoms (Kenney, 2003). *Self-esteem*, such as believing oneself to be worthy, was significantly associated with fewer distress symptoms (Thompson et al., 1996). *Perception of control* was linked to fewer depressive and anxiety symptoms; however, it was not associated with somatization nor hostility (Thompson et al., 1996). *Hope* and *trait forgiveness* were not significantly linked to distress symptoms (Tuck et al., 2012).

#### Cognitive Factors and Well-Being

For the subcategory Appraisals of the Traumatic Event, *meaning-making* was significantly linked to more posttraumatic growth (Johnson, 2021). *Forgiveness specific to the loss* was not significantly associated with well-being (Tuck et al., 2012). For the subcategory Cognitive Style, *thankfulness* was associated with more personal growth (Parappully et al., 2002). *Hope* and *trait forgiveness* were not significantly linked to well-being (Tuck et al., 2012).

### Emotional Factors and the Mental Health of HBI

Out of the 35 studies, two outlined that revenge had an impact on the mental health of HBI.

#### Emotional Factors and Distress

*Situational revenge* (e.g., motivation to seek revenge following a transgression; McCullough et al., 1998) was significantly linked to more CG and PTS symptoms (van Denderen et al., 2014). *Dispositional revenge* (i.e., attitudes toward vengeful personal responses to perceived wrongdoing; Stuckless & Goranson, 1992) was significantly linked to more CG symptoms; however, it was not associated with PTS symptoms (van Denderen et al., 2014). *Overall revenge* was not significantly linked to distress symptoms (Tuck et al., 2012).

#### Emotional Factors and Well-Being

*Situational revenge* was significantly linked to poorer functioning, although, *dispositional revenge* was not significantly linked to better functioning (van Denderen et al., 2014). *Overall revenge* was not significantly linked to well-being (Tuck et al., 2012).

### Other Factors and the Mental Health of HBI

Out of the 35 studies, 20 mentioned post-homicide factors that were not included in the categories based on the frameworks of Joseph et al. (1995) and Colquitt (2001). We refer to these as *Other factors*.

#### Other Factors and Distress

*Experiencing a high number of traumas since the murder* was significantly linked to greater distress symptoms (Thompson et al., 1998). Additionally, HBIs’ *satisfaction with services* was significantly associated with fewer depressive symptoms (Williams & Rheingold, 2015). Contradictory results were observed for *time since loss.* Eight studies reported that time since loss was not significantly associated with feelings of revenge, psychological maladjustment, PTS, distress, nor grieving symptoms (Amick-McMullan et al., 1989; Boelen et al., 2016; Burke et al., 2011; Sprang & McNeil, 1998; Thompson et al., 1996; Thompson et al., 1998; Tuck et al., 2012; van Denderen et al., 2014). In contrast, three studies demonstrated that time since loss was indeed associated with these factors (Soydas et al., 2021; van Denderen et al., 2016; van Wijk et al., 2017). *Working more hours* was linked to greater life satisfaction (Simmons et al., 2014). As for *perceived physical health*, Sprang and McNeil (1998) report that good physical health significantly predicts fewer grieving and PTS symptoms, though other researchers report conflicting findings. In fact, research by Williams et al. (2012) reveals that perceived physical health is not significantly linked to grieving, PTS, nor depressive symptoms.

#### Other Factors and Well-Being

*Time since loss* was significantly positively linked to greater posttraumatic growth (Johnson, 2021). *Time since loss* had no significant associations with well-being (Tuck et al., 2012). No study examined the association between *traumas experienced since the murder*, *employment status*, *satisfaction with services*, or *perceived physical health* and the well-being of HBI.

## Discussion

This is the first review, to our knowledge, to systematically synthesize the findings on the relationship between post-homicide factors and mental health among bereaved adults. Post-homicide factors, which refer to any factor which occurs after the homicide, were identified through database searches. Intervention-related factors were excluded as they had been previously reviewed by Alves-Costa, Hamilton-Giachritsis, Christie, et al. (2021). In total, 35 articles were included in the current review. Across these articles, the differing roles of a wide range of post-homicide factors are revealed. The most frequently documented categories included the CJS-related factors, social factors, and coping factors, followed by cognitive factors and emotional factors. Despite certain limitations, the current review provides mental health professionals with an organized compilation of the current findings in the literature, and could serve to inform practices in order to mitigate distress and promote well-being among HBI.

### Strengths and Limitations of Existing Research and Implications for Future Research

Most studies included in this review were published within the last 15 years (*n* = 27; 77.14%) suggesting that research on this topic is emerging rapidly. Of particular interest is the category of CJS-related factors (*n* = 18). One of the most prevalent and consistent factors in this category is the attendance of ongoing criminal proceedings, which has been repeatedly linked to greater distress symptoms. This suggests that certain interactions with the CJS could be harmful to HBI. Future research should focus on better understanding the dimensions of the CJS which exacerbate the distress of HBI. Another CJS-related factor that is associated with greater distress across a number of studies is the perception of a lack of information on behalf of the CJS. The current review revealed a gap in the current literature regarding the examination of distributive, procedural, and interpersonal perceptions of justice. In fact, perception of procedural justice has been associated with fewer PTS symptoms among crime victims (Wemmers, 2013). Given our current knowledge that the perception of fairness in HBI could serve to reduce their anxiety (Van den Bos & Lind, 2002), it appears crucial to deepen our understanding of the role of distributive, procedural, and interpersonal perception of justice in the mental health of HBI. Furthermore, the current literature reveals contradictory results regarding satisfaction with the CJS. While some studies suggest that there is a link between healing and satisfaction with the CJS, others do not support this association. The systematic review of Kunst et al. (2015), conducted on crime victims, also revealed mixed results regarding the association between satisfaction with the CJS and emotional recovery. This discrepancy could be explained by methodological issues, such as the use of differing assessment tools for the perception of justice and satisfaction with the CJS, which are often closely intertwined in the current literature. Shedding light on what distinguishes these two dimensions is crucial.

Various posttraumatic factors which have been identified as significant predictors in other populations have not yet been measured in HBI. For example, no study has examined the association between the assumption world views, or self-efficacy of HBI with their mental health, though associations have been established with different types of victims (Cieslak et al., 2008; Matthews & Marwit, 2004). Losing a loved one to homicide has the potential to profoundly disrupt fundamental beliefs about life, society, the world, justice, or human nature (Janoff-Bulman, 1989); in turn, understanding the role of this factor is crucial in fostering positive outcomes in the mental health of the bereaved.

Few studies have examined the positive dimensions of mental health, such as well-being, of HBI in relation to post-homicide factors. Of those who have, results indicate that the holding of a verdict, engaging in meaning-making, maintaining a bond with the victim, and not drinking alcohol were associated with greater posttraumatic growth, joy, and life satisfaction. However, the associations between well-being and other factors, such as satisfaction with the CJS, perceived social support, and negative cognitions, have yet to be studied. Considering the current operationalization of mental health as not only the absence of psychopathological symptoms, but also as the presence of positive psychological states (e.g., Massé et al., 1998), future studies should examine the determinants of positive mental health indicators among HBI.

Furthermore, inconsistencies concerning certain factors have been observed in the existing research. Of particular importance is the relationship between *social support* and grieving symptoms. As expected, a negative association was observed between perceived social support and grieving symptoms, in a study by Sprang and McNeil (1998). However, a different study (Burke et al., 2010) reported a non-significant relationship between perceived grief support and grieving symptoms. This suggests that HBI might benefit more from general social support than from support specific to their grief. Additionally, greater need for physical assistance was unexpectedly associated with more grieving symptoms in a study by Bottomley et al. (2017). It is possible that the high level of distress, or the high needs of HBI, lead to a depletion of resources on behalf of the HBIs’ sources of social support, as suggested by Bottomley et al. (2017). The supporters’ exhaustion could further lead to them to withdraw or become unavailable. In turn, the social support received by HBI might not be in line with their needs or desires, which could decrease their well-being (e.g., Siewert et al., 2011). These findings highlight the need for future studies to investigate the discrepancies between desired support (or the need for support) and support received.

Most studies included in our review are cross-sectional, which prevents our ability to make inferences about causality and the direction of the relationship between post-homicide factors and mental health dimensions. In fact, only seven studies included multiple time points. In the study of Burke et al. (2011), CG symptoms at the first time point predicted more negative religious coping at the second time point. Therefore, future studies should opt for longitudinal designs to highlight the bidirectional relationship between post-homicide factors and mental health.

The AXIS tool was applied to assess the risk of bias. Most studies had a large sample size, which represents an important methodological strength. However, the quality of the studies varied significantly, suggesting that the results of studies with high risk of bias should be interpreted with caution. More specifically, sampling biases, such as most participants being recruited from community-based organizations aimed at helping them cope with their loss, may lead to a sample of HBI that are more psychologically distressed than those who are not seeking care. In turn, results might not be representative of the population of HBI not seeking mental health services and should be interpreted with caution. Other methodological concerns include the measurement of post-homicide factors and mental health dimensions. Most of the included studies did not use structured clinical interviews but rather self-report measures (some developed assessment tools specifically for their study), which could decrease the validity of the result obtained as well lead to an overestimation of the symptoms reported by HBI (Thombs et al., 2018). Future studies should favor clinician-administered interviews or validated instruments such as the PCL-5 (Weathers et al., 2013) and the Organizational Justice Scale (Colquitt, 2001).

### Strengths and Limitations of the Systematic Review

This review systematically included both positive and negative dimensions of mental health, as recommended by dual-continua or dual-factor models (e.g., Massé et al., 1998). This constitutes an important strength of our current review and distinguishes it from reviews conducted previously (e.g., Tankersley et al., 2021). Also, the inclusion of different types of research designs allows for a broader understanding of the lived experience of HBI. However, the heterogeneity of research designs and of mental health measures made comparisons difficult and could explain some of the mixed findings. As such, it was more appropriate to conduct a narrative synthesis than a meta-analysis. In an effort to reduce the risk of publication bias, our review includes studies that did not find a significant relationship between post-homicide factors and mental health indicators. Results also suggest good inter-rater reliability scores in the risk of bias assessments. However, articles were not excluded according to their score on the AXIS, constituting an important consideration for the interpretation of the results of studies with higher risk of bias.

In order to be included in this review, articles could only include HBI, which lead to the exclusion of many studies with heterogenous samples of bereaved individuals (e.g., those bereaved by violent death including homicide, suicide, or accident). In the current literature, considerable discrepancies are observed between the experiences of HBI and those bereaved by other types of deaths, in terms of their cognitions and the intensity of their distress (Murphy et al., 2003). Therefore, focusing only on HBI allowed for a unique focus on factors specific to their mental health. However, we encourage future research to focus on the differences between HBI and other individuals bereaved in terms of their mental health and post-loss factors.

### Clinical Implications and Conclusion

This review could help mental health workers prepare a more adequate treatment plan for reducing the distress and improving the well-being of HBI. Addressing significant harmful factors, such as the painful experience of HBI with the CJS and their resulting perceptions of injustice, could be validating and beneficial for the bereaved. Significant post-homicide factors related to resilience, such as meaning-making and anger management*,* could be utilized advantageously in counselling. For example, mental health workers could focus on helping HBI integrate their experience of the loss into their existing understanding of themselves, others, and the world to help them find a certain level of serenity and purpose in their suffering (Pearlman et al., 2014). Mental health professionals could also use cognitive restructuring to promote flexibility in HBI’s assumptions about the meaningfulness of the world (Resick et al., 2016). Training the CJS personnel about the characteristics of this population appears crucial. For instance, implementing training programs that encourage CJS personnel to consider the needs of HBI and communicate with them in a fair, appropriate, and sensitive manner could be crucial. Recommendations such as these could be made to the CJS to better support HBI in their court attendance. Finally, although many mental health workers are reluctant to address religious beliefs, the scientific literature indicates that they may significantly impact post-homicide distress and should therefore be included in the therapeutic process.

## Supplemental Material

Supplemental Material - Mental Health of Homicidally Bereaved Individuals: A Systematic Review of Post-Homicide Factors Supplemental Material for Mental Health of Homicidally Bereaved Individuals: A Systematic Review of Post-Homicide Factors by Sarah Lebel, Olivier Lépine, and Pascale Brillon in OMEGA - Journal of Death and Dying
