# Supplementary material for: Mental Health of Homicidally Bereaved Individuals: A Systematic Review of Post-Homicide Factors
Source: Omega (Westport). 2024 Apr 7;93(1):659–88. doi: 10.1177/00302228241245751 (PMC13002928; doi:10.1177/00302228241245751)
Supplement: Supplemental Material - Mental Health of Homicidally Bereaved Individuals: A Systematic Review of Post-Homicide Factors [file sj-pdf-1-ome-10.1177_00302228241245751.pdf]

Supplemental Material

Table 1

Risk of Bias for Each Study (n = 35)

#	First author and year	Introduction (out of 1) Item 1	Method (out of 10) Items 2 to 11	Results (out of 5) Items 12 to 16	Discussion (out of 2) Items 17 and 18	Other (out of 2) Items 19 and 20	Total score (out of 20)
1	Alves-Costa (2018)	1	8	4	2	1	16
2	Alves-Costa (2021)	1	8	4	2	1	16
3	Amick- McMullan (1989)	1	4	4	2	1	12
4	Baliko (2008)	1	8	4	2	2	17
5	Boelen, van Denderen & de Keijser (2016)	1	10	3	2	2	18
6	Bottomley (2017)	1	8	2	1	2	14
7	Burke (2010)	1	8	2	2	1	14
8	Burke (2011)	1	9	4	2	1	17
9	Hannays-King (2015)	1	7	3	0	0	11
10	Johnson (2021)	1	9	4	2	2	18
11	Kenney (2003)	1	4	2	0	0	7
12	McDevitt-Murphy (2012)	1	9	3	2	1	16
13	McDevitt-Murphy (2021)	1	9	5	2	2	19
14	Milman (2018)	1	8	4	2	1	16
15	Neimeyer (2011)	1	9	4	2	1	17
16	Parappully (2022)	1	8	3	0	0	12
17	Rheingold & Williams (2015)	1	9	4	2	1	17
18	Rynearson (1995)	1	7	2	0	0	10
19	Simmons (2014)	1	5	2	2	1	11
20	Soydas (2020)	1	9	4	2	2	18
21	Soydas (2021)	1	8	3	2	2	16
22	Sprang (1998)	1	7	3	1	0	12
23	Stretesky (2010)	1	8	5	2	1	17
24	Thiel (2016)	1	6	4	0	1	12
25	Thompson (1996)	1	8	3	2	0	14
26	Thompson (1997)	1	8	3	1	0	13
27	Thompson (1998)	1	8	3	2	0	14
28	Tuck (2012)	1	9	3	2	2	17
29	Van Wijk (2017)	1	8	3	1	1	14
30	van Denderen (2016)	1	8	3	2	2	16
31	van Denderen (2014)	1	9	3	2	1	16
32	Wellman (2014)	1	9	4	2	1	17
33	William (2012)	1	9	4	2	1	17
34	William (2015)	1	10	3	2	1	17
35	Zakarian (2019)	1	8	2	2	1	14
